# Intention to use telemonitoring for chronic illness management and its associated factors among nurses and physicians at public hospitals in Bahir Dar, northwest Ethiopia: using a modified UTAUT2 model

**DOI:** 10.3389/frhs.2025.1460077

**Published:** 2025-03-06

**Authors:** Temesgen Ayenew Alameraw, Mulusew Andualem Asemahagn, Kassahun Dessie Gashu, Agmasie Damtew Walle, Jenberu Mekurianew Kelkay, Abebaw Belew Mitiku, Geleta Nenko Dube, Habtamu Alganeh Guadie

**Affiliations:** ^1^ Department of Health Informatics, School of Public Health, College of Medicine and Health Sciences, Arba Minch University, Arba Minch, Ethiopia; ^2^ Department of Health System Management and Health Economics, School of Public Health, College of Medicine and Health Sciences, Bahir Dar University, Bahir Dar, Ethiopia; ^3^ Department of Health Informatics, Institute of Public Health, College of Medicine and Health Sciences, University of Gondar, Gondar, Ethiopia; ^4^ Department of Health Informatics, College of Health Sciences, Mettu University, Mettu, Ethiopia; ^5^ Department of Health Informatics, School of Public Health, College of Medicine and Health Sciences, Dilla University, Dilla, Ethiopia; ^6^ Department of Health Informatics, Arba Minch College of Health Sciences, Arba Minch, Ethiopia

**Keywords:** intention to use, telemonitoring, UTAUT, chronic patients, Ethiopia

## Abstract

**Background:**

Patients with chronic illnesses need to take care of themselves and seek ongoing medical attention. By using technology, telemonitoring can minimize hospitalization and care costs, while increasing professional productivity, providing constant medical attention and enhancing patient self-care management. Despite all these advantages, nothing is known regarding the intentions of Ethiopian professionals and nurses to adopt telemonitoring technologies. Therefore, the purpose of this study is to evaluate the telemonitoring intention of Ethiopian professionals and nurses, as well as the factors related to it.

**Methods:**

A total of 781 randomly chosen nurses and physicians who worked at public hospitals in Bahir Dar City, northwest Ethiopia, participated in a cross-sectional survey. To give everyone an opportunity, the sample size was distributed equitably among the hospitals and the profession according to staffing numbers. The sample was obtained using a simple random sampling technique. Data were gathered by skilled data collectors utilizing a self-administered questionnaire. For additional cleaning and descriptive statistical analysis, the data were imported into EpiData version 4.6 and exported to Statistical Package for Social Science version 25. Analysis of Moment Structure version 23 structural equation modeling was used to ascertain the degree of the association between the variables.

**Result:**

The response rate was 732/781 (93.7%), with 55.7% (408/732) of the participants being men and two-thirds (67.6%, 495/732) being nurses. About 55.9% [95% confidence interval (CI): 52.3–59.6] of respondents intended to use telemonitoring. The desire to employ telemonitoring is positively impacted by performance expectancy (*β* = 0.375, 95% CI: 0.258–0.494), effort expectancy (*β* = 0.158, 95% CI: 0.058–0.252), facilitating condition (*β* = 0.255, 95% CI: 0.144–0.368), and habit (*β* = 0.147, 95% CI: 0.059–0.233). Age and gender positively affected the link between effort expectancy and intention to employ telemonitoring. It was discovered that being young and male has a beneficial relationship impact. Age positively moderated the association between the intention to use telemonitoring and the facilitating conditions, and adults were strongly linked with the relationship.

**Conclusion:**

In Bahir Dar City public hospitals, over half of the doctors and nurses have the intention to use telemonitoring. Predictive indicators of intention to utilize telemonitoring that were statistically significant were performance expectancy, effort expectancy, facilitating condition, and habit.

## Introduction

Telemedicine refers to the remote delivery of healthcare services, including consultations, diagnosis, and treatment, using telecommunications technology (1, 2). Telemonitoring (TM) is a key component of telemedicine (3). Telemonitoring is remotely monitoring vital parameters by individuals outside of a healthcare setting and electronically transmitting them to healthcare providers (4–7). It remotely monitors the health state of patients using audio, video, and other telecommunications and electronic information processing technologies (8). Telemonitoring systems (TMS) are increasingly implemented for the follow-up of chronic diseases because they improve the quality of healthcare and allow capturing the clinical parameters of patients easily and continuously (9).

Currently, chronic diseases are significant global, national, and personal health problems (10) that require continuous medical attention as well as patient self-management. However, due to geographic obstacles and inadequate access to medical personnel, the rate of prompt and ongoing follow-up with chronic patients in Ethiopia stayed low (11, 12). These obstacles have been overcome with the use of telemonitoring systems (13).

TM technologies have the potential for enhancing the quality of life, raising the general standard of living and services and better managing common conditions, complications, life-threatening events, and mortality (14, 15). Among digital health solutions for health workers establishing remote patient monitoring services/programs at public hospitals is a strategic initiative planned by the Ministry of Health of Ethiopia (16). However, there is limited information on the intention to use telemonitoring (IUTM) among nurses and professionals in Ethiopia.

Numerous researchers have shown that healthcare professionals around the world have little intention of using telemonitoring technologies (15). The problem is getting worse in Africa due to high technology resistance from healthcare providers as a result of technology anxiety (17, 18). A study conducted in Ethiopia showed low willingness to use telemedicine (46.5%) (19).

A low intention to employ telemonitoring technology results in a limited uptake of telemonitoring systems for monitoring chronic illnesses using telemonitoring (20). The consequences of low adoption of telemonitoring systems are a high hospitalization rate, increased cost of care, inhibited self-care management, and low healthcare access, which leads to poor management of chronic disease and increased burden of diseases, disability, and premature death (17, 21). A number of studies have revealed that performance expectancy (PE), effort expectancy (EE), social influence (SI), facilitating condition (FC), hedonic motivation (HM), and habit (HA) (20, 22–24) are determining the status of intention to use telemonitoring among nurses and physicians.

According to my exhaustive search, there are a few studies that are known to be conducted outside of Ethiopia with the intention to use telemonitoring. These studies have drawbacks, including outdated research, small sample sizes, disagreement gaps, and bias toward participation (20, 24). Therefore, the present study aims to assess the intention of nurses and physicians to use telemonitoring and associated factors to support chronic patients among medical professionals and nurses employed at Bahir Dar City's public hospitals.

Prior studies have demonstrated that the use of telemonitoring system implementation depends on the intention of the user. As a result, it is crucial to conduct studies on the intention of health professionals to use telemonitoring systems (24–26). Therefore, knowing the intention to use telemonitoring and its challenges among health workers will be important to patients with chronic diseases, program owners, policymakers, and researchers to take evidence-based interventions and conduct further studies to support prevention, diagnosis, and treatment follow-up.

### Theoretical background and hypothesis

The Unified Theory of Acceptance and Use of Technology (UTAUT) was developed in 2003 by Venkatesh et al. (22), and it was updated in 2012 by Venkatesh et al. (23). The UTAUT is used to better understand user acceptance of technology and how technology design might influence consumer decisions to accept or reject it. This model was derived from seven other theoretical models: the Theory of Reasoned Action (TRA), the Theory of Planned Behavior (TPB), the Technology Acceptance Model (TAM), the Motivational Model (MM), the Model of PC Utilization (MPCU), the Combined TAM and TPB (C-TAM-TPB), and the Innovation Diffusion Theory (IDT) (22).

To provide answers to research questions, it is essential to use the right model as a theoretical foundation to best describe consumer behavior toward the technology under consideration. The UTAUT2 model was chosen as the main theoretical framework for the inquiry of this study. According to information systems research, the UTAUT had a high explanatory power and was remarkably successful in evaluating the variables that affect a person's intention to utilize various technologies (27).

**Figure 1:**
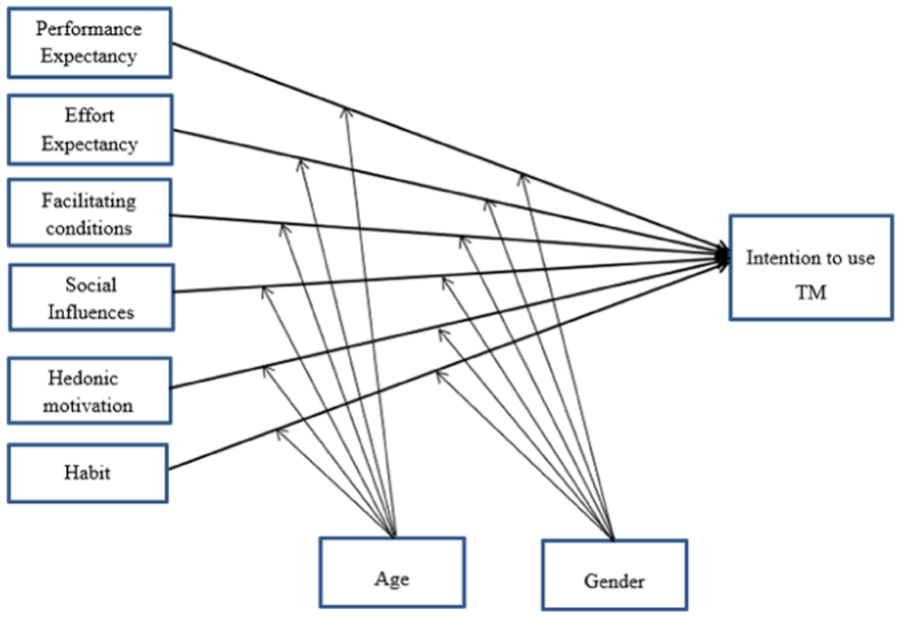
Proposed theoretical model. Adapted with permission from “Research Model UTAUT2” by Viswanath Venkatesh, James Y. L. Thong and Xin Xu, licensed under CC BY 4.0.

In this study, the UTAUT2 model was proposed by removing the experience from the moderator variable, price value from the exogenous variable, and actual use from the endogenous variable from the original UTAUT2 model. Because telemonitoring technology has not yet been fully implemented throughout Ethiopia, nurses and physicians have not yet fully utilized the technology. The actual use behavior that was an endogenous variable in the original UTAUT2 model will not be measured in this study. Likewise, the technology is not used in Ethiopia; as a result, no moderator was included with experience because nurses and doctors there are not familiar with TM. In the case of resources to adopt TM, which was fulfilled by the government price value is not the concern of nurses and physicians; thus, the price value was not included as an exogenous variable. Therefore, the current proposed UTAUT2 model constructs of intention to use telemonitoring are performance expectancy, effort expectancy, social influence, facilitating conditions, hedonic motivation, and habit, and two moderator variables, age and gender (23) (Figure 1).

### Performance expectancy

PE is “the degree to which an individual believes that using the system will help him or her to gain in job performance” (22). The UTAUT models capture the concept of performance expectancy from the constructs of five models, perceived usefulness (TAM/TAM2 and C-TAM-TPB), extrinsic motivation (MM), job fit (MPCU), relative advantage (IDT), and outcome expectations [standardized clinical trial (SCT)] (22, 28, 29). In Bilbao Primary Care (20) and Don Ostia University Hospital (24) in Spain, it has been found that PE has a positive association with the intention of using telemonitoring. In Bangladesh (30) and Ethiopia (31), PE has a significant association with the intention of using electronic health (eHealth). In India (27), PE greatly influences behavioral intention to employ the suggested mobile-based information technology (IT) solution. A comparable study conducted in Ethiopia showed that PE had no direct association with the intention to use telemedicine. Therefore, to test the effect of PE on the intention to use TM, the following hypothesis is generated:

H1: The intention to use TM will be positively influenced by performance expectations.

### Effort expectancy

Effort expectancy is “the degree of ease associated with the use of the system” (22)*.* UTAUT models capture the concept of effort expectancy from the perceived ease of use (TAM/TAM2), complexity (MPCU), and ease of use (IDT) (22, 28, 29). A systematic review conducted among 12 studies showed that effort expectancy was found to be the strongest predictor of technology acceptance (28). Studies conducted in (20)Bilbao Primary Care showed that EE had a positive association with the intention to use telemonitoring. A study conducted in India (27) showed that EE was found to significantly influence behavioral intention to use the proposed mobile-based IT solution. Another study conducted in Ethiopia (32) and Indonesia (33) showed that EE significantly affects the intention to use telehealth/telemedicine. In a comparable study conducted in Don Ostia University Hospital (24) in Spain, it was found that EE did not influence the intention to use telemonitoring. Therefore, to test the effect of EE on the intention to use TM, the following hypothesis is generated.

H2: The intention to use TM will be positively affected by effort expectations.

### Social influences

Social influence is “the degree to which an individual perceives that important others believe he or she should or should not use the new system” (29, 34). Social influence as a direct determinant of intention to use is represented as the subjective norm in TRA, TAM2, TPB/dual theory of planned behavior (DTPB), and C-TAM-TPB, social factors in MPCU, and image in IDT (22). Studies conducted in Bilbao Primary Care (20) and Don Ostia University Hospital (24) showed social influences were not positively associated with the intention to use telemonitoring. Another study conducted in Indonesia (33) and Ethiopia (32) showed that social influence is not significantly associated with the intention to use telehealth. Comparable studies conducted in the original UTAUT model (22) showed a strong association with the intention to use information technology. Similarly, in studies conducted in Taiwan (35) and Senegal (36), it was found that social influence had a strong association with the intention to telemedicine. In another study conducted in India (27), SI was found to significantly factor in the intention to use the proposed mobile-based IT solution. Therefore, to test the effect of SI on the intention to use TM, the following hypothesis is generated.

H3: The intention to use TM will be positively affected by social influences.

### Facilitating conditions

Facilitating conditions is “the degree to which an individual believes that an organizational and technical infrastructure exists to support the use of the system” (22, 29). This definition captures constructs from three models perceived, behavioral control [theory of planned behavior (TBP) DTPB, C-TAM-TPB], facilitating conditions (MPCU), and compatibility (IDT). A study done in Bilbao Primary Care (20) and Don Ostia University Hospital (24) showed that facilitating conditions are a significant predictor of intention to use telemonitoring. Similarly, studies conducted in Indonesia (33) and Ethiopia (31) indicate that facilitating conditions are strongly associated with the use of eHealth/telehealth technologies. Another study conducted in India (27) showed that FC was found to significantly influence the intention to use the proposed mobile-based IT solution. Comparable studies conducted in Ethiopia (32) showed that facilitating conditions did not have a significant influence on the intention to use telemedicine. Therefore, to test the effect of FC on the intention to use TM, the following hypothesis is generated:

H4: The intention to use TM will be positively affected by facilitating conditions.

### Hedonic motivation

Hedonic motivation is described as the extent to which a person thinks funny or enjoyable due to the employment of a particular technology (23). The modified UTAUT showed that hedonic motivation has a positive influence on behavioral intention (23). Studies conducted in China (37) and Pakistan (38) showed that hedonic motivation had a significant effect on the intention to use telemedicine. A study conducted in the United States showed that hedonic motivation had a significant effect on mobile-based app acceptance (39). A study conducted in Malaysia showed that HM had a significant effect on intention toward smartwatches for health and fitness monitoring (40). Studies conducted in Italy revealed that hedonic motivation was a significant factor in the use of robot technologies (41). Therefore, to test the effect of HM on the intention to use TM, the following hypothesis is generated:

H5: The intention to use TM will be positively affected by hedonic motivation.

### Habit

Habit refers to automating behavior from initial learning to regular use of technology (23). A study conducted in the United States showed that habit had a significant effect on mobile-based app acceptance (39). Therefore, to test the effect of HB on the intention to use TM, the following hypothesis is generated:

H6: The intention to use TM will be positively affected by habit.

### Moderator variables of intention to use TM

In the UTAUT2 model of context, sex, age, and experience variables affect the direction or strength of the relation between exogenous and endogenous variables (42).

### The moderating effect of age

In a study conducted in China, a moderator analysis confirmed that different age groups have specific moderating effects on effort expectancy and behavioral intention to use health technology (43). In another study conducted in the Midwestern US state, it was found that the effects of PE, EE, SI, HA, and HM on behavioral intention to use health information technology were all moderated by individual age (23). In another study conducted in Asia, PE, EE, and SI were moderated by age to behavioral intention to use smart equipment for health (44). Studies in the original model of the UTAUT showed that age had moderating effects on PE, EE, and SI on behavioral intention to use information technology (22). Therefore, to test the moderating effect of age on the intention to use TM, the following hypotheses are proposed:

H7: The influence of PE on the intention to use TM will be moderated by age.

H8: The influence of EE on the intention to use TM will be moderated by age.

H9: The influence of FC on the intention to use TM will be moderated by age.

H10: The influence of SI on the intention to use TM will be moderated by age.

H11: The influence of HM on the intention to use TM will be moderated by age.

H12: The influence of HA on the intention to use TM will be moderated by age.

### The moderating effect of gender

A study conducted in China indicated that gender had moderating effects on EE on behavioral intention to use the health system (43). Studies in the original model of the UTAUT showed that gender had moderating effects on PE, EE, SI, and FC on behavioral intention to use information technology (22). Another study conducted in India showed that EE had a moderating effect on the intention to use mobile-based Information Technology (27). Similarly, in a study conducted in the Midwestern US state, the effects of PE, EE, SI, HA, and HM on behavioral intention to use health information technology were all moderated by individual gender (23). Therefore, to test the moderating effect of gender on the intention to use TM, the following hypotheses are proposed:

H13: The influence of PE on the intention to use TM will be moderated by gender.

H14: The influence of EE on the intention to use TM will be moderated by gender.

H15: The influence of FC on the intention to use TM will be moderated by gender.

H16: The influence of SI on the intention to use TM will be moderated by gender.

H17: The influence of HM on the intention to use TM will be moderated by gender.

H18: The influence of HA on the intention to use TM will be moderated by gender.

## Methods

### Study design and setting

A facility-based cross-sectional study was conducted from 25 March to 30 April 2023, at Bahir Dar city public hospitals in northwest Ethiopia Amhara region [Addis Alem Primary Hospital (AAPH), Felege Hiwot Comprehensive Specialized Hospital (FHCSH), and Tibebe Ghion Specialized Teaching Hospital (TGSTH)]. These public hospitals have a total of 2,566 health professionals. Out of them, 1,112 were nurses and physicians who were permanently employed. Bahir Dar is the capital city of the Amhara National Regional State located at a distance of 565 km northwest of Addis Ababa, the capital city of Ethiopia. Amharic is the working language of the state. According to the Amhara Bureau of Finance and Economic Development (ABOFED), the population of Bahir Dar city was estimated to be 339,683. Among these, 183,307 (54%) of them were women (45). The city had 11 health centers (including one private health center), 10 health posts and one family guidance association clinic, 4 private general hospitals, and 35 medium private clinics.

### Study participants and sample size determination

All physicians and nurses working at AAPH, FHCSH, and TGSTH are the source population for this study. All physicians and nurses working at AAPH, FHCSH, and TGSTH during the study period are the study population for this study. The study included all physicians and nurses working at AAPH, FHCSH, and TGSTH. However, the physicians and nurses who are not permanently employed (less than 6 months) in AAPH, Felege Hiwot comprehensive specialized hospital (FHRH), and TGSTH were excluded since they may not have the knowhow about predictors’ intention to use TM. The sample size was determined based on the free parameters assumption as follows.

### Model specification

Model specification is a visual representation of theoretical variables of interest and expected relationships among them, as well as an expression of hypotheses with graphical conceptual models (46). The exogenous variables (PE, EE, SI, FC, HM, and HA) are factors of TM.

### Model identification

Based on paths, the following rules for determining model parameters can be estimated using a diagram (47). All variances of the exogenous and endogenous variables, including disturbance, are free parameters (25 error terms, 1 disturbance, and 6 exogenous latent variables, totaling 32). All covariances between exogenous variables are free parameters (15 covariances between exogenous variables). All factor loadings between latent and its indicators are free parameters (18 load factors between latent and its indicators without considering a fixed load factor). All regression coefficients between observed and latent variables are free parameters (six regression coefficients between exogenous and endogenous). The total number of free parameters in this model is 71. The number of distinct parameters (sample moment) to estimate is k(k + 1)/2 = 25 × 26/2 = 325, where k is the number of items in our study. Degree of freedom = sample moment − free parameters = 325 − 71 = 254.

The model has been overidentified, given that there are more than 0 degrees of freedom. Working with an overidentified model is, therefore, typically recommended (46, 48). The number of free parameters in the hypothetical model determines the minimum sample size; it has been suggested that the ratio of free parameters to respondents be estimated as 1:10 (49). Accordingly, considering the 71 free parameters to be estimated based on the hypothesized model and considering participants to be estimated as a free parameter ratio of 10, the minimum sample required is 710. The sample size calculated accounts for the non-response rate of 10% and is therefore considered to demonstrate the final sample size. Thus, the final sample size becomes 781.

$$\begin{array}{c} \mathrm{Mathematically}:\;\mathrm{Fn}=(\mathrm{FP}\times R)+\mathrm{nr}\\ \;\mathrm{Fn}=(71\times 10)+(71\times 10)\times 10\text{\%}\\ \;\mathrm{Fn}=781 \end{array}$$

where Fn is the final sample size, FP is the free parameter to be estimated, R is the number of individuals to be selected for each free-estimable parameter, and nr is the non-response rate using 10%.

### Sampling procedure

A proportional allocation of participants for each public hospital was done. Then, the participants were allocated based on their profession. Finally, 781 participants were selected by a simple random sampling method using OpenEpi random program version 3 from their respective professions.

### Data collection tools and procedures

A structured self-administered questionnaire was developed after reviewing several works of literature on the subject (20, 23, 24, 27, 32, 50). The structured questionnaire had two parts: the first part contains sociodemographics with 7 items, and the second part contains 25 items of questions from key constructs of the UTAUT model. Four performance expectation items, four effort expectancy items, four facilitating condition items, three social influence items, three hedonic motivation items, four habit items, and three intention to use TM items are all included in the tool. A Likert scale with values ranging from 1 to 5 was used to measure each of the constructs: 1 representing strongly disagree, 2 disagree, 3 neutral, 4 agree, and 5 highly agree. Amharic language questions were used because Amharic is the common local language in the study area. Therefore, it is necessary to translate the original English version of the questions into Amharic. The back-translation method was utilized to translate from English into Amharic with the same meaning and originality in both versions and then translate the questions back into English. This step was important to ensure that the participants would not be misunderstood because of language barriers. A case scenario was prepared for nurses and physicians who support chronic patients, who might not be aware about telemonitoring during the data collection period.

After the final data collection tool was provided to trained data collectors, they collected data after giving detailed information about the study and getting consent from participants. Data collection was fully voluntary, including the right to withdraw from the study at any time.

### Data quality control

Even though a questionnaire was developed from standardized tools, it was pretested by taking 5% of the total sample size of physicians and nurses, done at Finote Selam Hospital before data collection. Then, necessary correction was done based on the pretest finding. Two-day training was given to three data collectors and two supervisors about the study's purpose, data collection techniques, data collection tools and procedures, respondent approach, data confidentiality, and respondent rights. Supervisors and investigators examined the completeness and consistency of data regularly. After data collection, the data were cleaned and missing values were managed properly.

### Data processing and analysis

EpiData version 4.6 was used to enter the data into the computer. Then, it was exported to Statistical Package for Social Science (SPSS) version 25 for additional examination. To characterize the dependent and additional research variables, descriptive statistics were calculated. Using Analysis of Moment Structure (AMOS) version 23, an SPSS add-in program, the measurement model was evaluated by confirmatory factor analysis (CFA), and the hypothesis was assessed by structural equation model (SEM) analysis. The assumption of a multivariate outlier was checked using Mahalanobis d^2^, and data normality was assessed by multivariate kurtosis <5, and critical ratio for skewness between −1.96 and 1.96. Multicollinearity was assessed using variance inflation factors (VIFs) <10 and tolerance >0.1. A correlation between constructs and factor loadings for each item was tested; therefore, the value of factor loading for each item should be more than 0.6 (51).

The model's goodness of fit was assessed using the following metrics: Chi-square ratio (>0.9), the goodness-of-fit index (GFI > 0.9), adjusted goodness-of-fit index (AGFI > 0.8), root mean square error approximation (RMSEA < 0.08), and root mean square of the standardized residual (RMSR < 0.088) (49). If the model fit indices were below the cutoff point or there was model misspecification, either the item that was below the cutoff point was deleted or a high value of modification indices was used to enhance model fit indices until the model was fitted with a threshold value of maximum four times (51).

Construct reliability and validity were evaluated to determine the extent to which a variable or combination of variables was consistent in what it wanted to measure and to evaluate how effectively the selected construct item measured the construct. Construct reliability was obtained by calculating composite reliability above the required level of 0.70 (52).

Convergent validity was determined using the average variance extracted (AVE) method, with values above the 0.50 threshold. The Fornell and Larcker criterion, as well as the Heterotrait–Monotrait (HTMT) ratio, was used to determine discriminant validity. It was determined whether the square root of AVE of less than 0.9 for a construct was greater than its correlation with the other constructs in the study (51). The value of the HTMT ratio required a limit of less than 0.8 (52).

The standardized path coefficient and variances were used to measure the relationship between exogenous and endogenous variables, as well as 95% confidence intervals (CIs) and *p*-value to determine statistical significance (*p*-value < 0.05).

The moderator can be a continuous or categorical variable, and it can alter the relationship between independent and dependent variables through interaction effects and multiple group analysis (32). Because gender (male, female) and age (below 30 years, above or equal to 30 years) (53) are categorical, the moderating effects of predictors among the hypothesized paths within the core research model were tested using multiple group analysis. The chi-square difference (change in Χ^2^) and *p*-value between unconstrained and constrained (structural weight) models were estimated to determine the effect of the moderator.

## Results

### Sociodemographic characteristics of nurses and physicians

In this study, from the expected 781 responses, 732 (93.7%) response rates of nurses and physicians working at public hospitals in Bahir Dar were reported. Half (50.1%, 367/781) of the participants were from Felege Hiwot Comprehensive Specialized Hospital, and 55.7% (408/732) of the participants were male. The median age of the respondents was 35 [interquartile range (IQR): 30–38] years. Two-thirds (67.6%, 495/732) of the participants were nurses and 69% had a first degree (Table 1).

**Table 1:** Sociodemographic characteristics of nurses and physicians at public hospitals in Bahir Dar northwest Ethiopia, 2023.

Sociodemographic characteristics	Categories	Frequency (*n*)	Percent
Institutions	AAPH	34	4.6
	FHCSH	367	50.1
	TGSTH	331	45.2
Gender	Female	324	44.3
	Male	408	55.7
Age group (years)	<30	167	22.8
	30–39	444	60.7
	40–49	108	14.8
	>49	13	1.8
Profession	Nurse	495	67.6
	General practitioners	95	13.0
	Specialist physician	133	18.2
	Subspecialist physician	9	1.2
Education level	Diploma	42	5.7
	First degree	505	69
	Second degree	175	23.9
	Third degree	10	1.4
Experiences (years)	Less than 5	196	26.9
	Between 5 and 10	403	51.1
	Above 10	132	18
Average monthly income (ETB)	Below 5,000	8	1.1
	5,000 to <10,000	455	62.2
	10,000–15,000	252	34.4
	Above 15,000	17	2.3

### Intention to use telemonitoring among nurses and physicians

**Figure 2:**
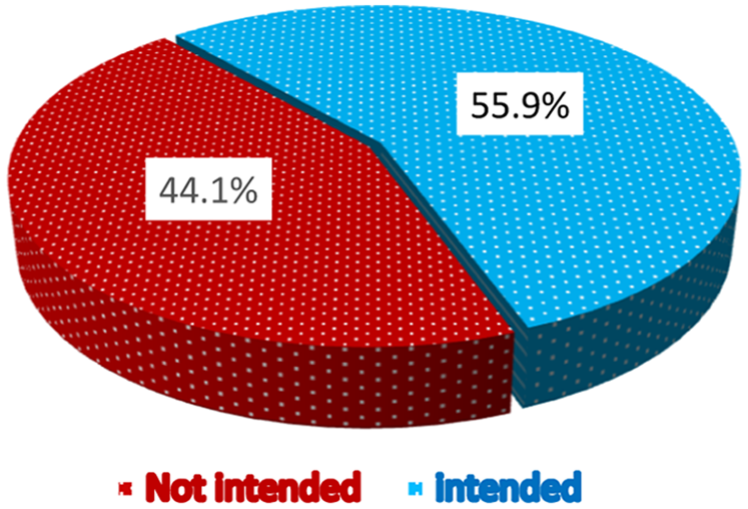
Proportions of intention to use telemonitoring among nurses and physicians at public hospitals in Bahir Dar northwest Ethiopia, 2023.

In this study, over half (55.9%, 95% CI: 52.3–59.6) of the study participants had the intention to use telemonitoring to support chronic patients. The median score of computed three indicators with five Likert scale of the intention to use a telemonitoring system was 11, with a standard deviation of 3.229, and the minimum and maximum scores were 3 and 15, respectively (Figure 2).

### Measurement model

**Figure 3:**
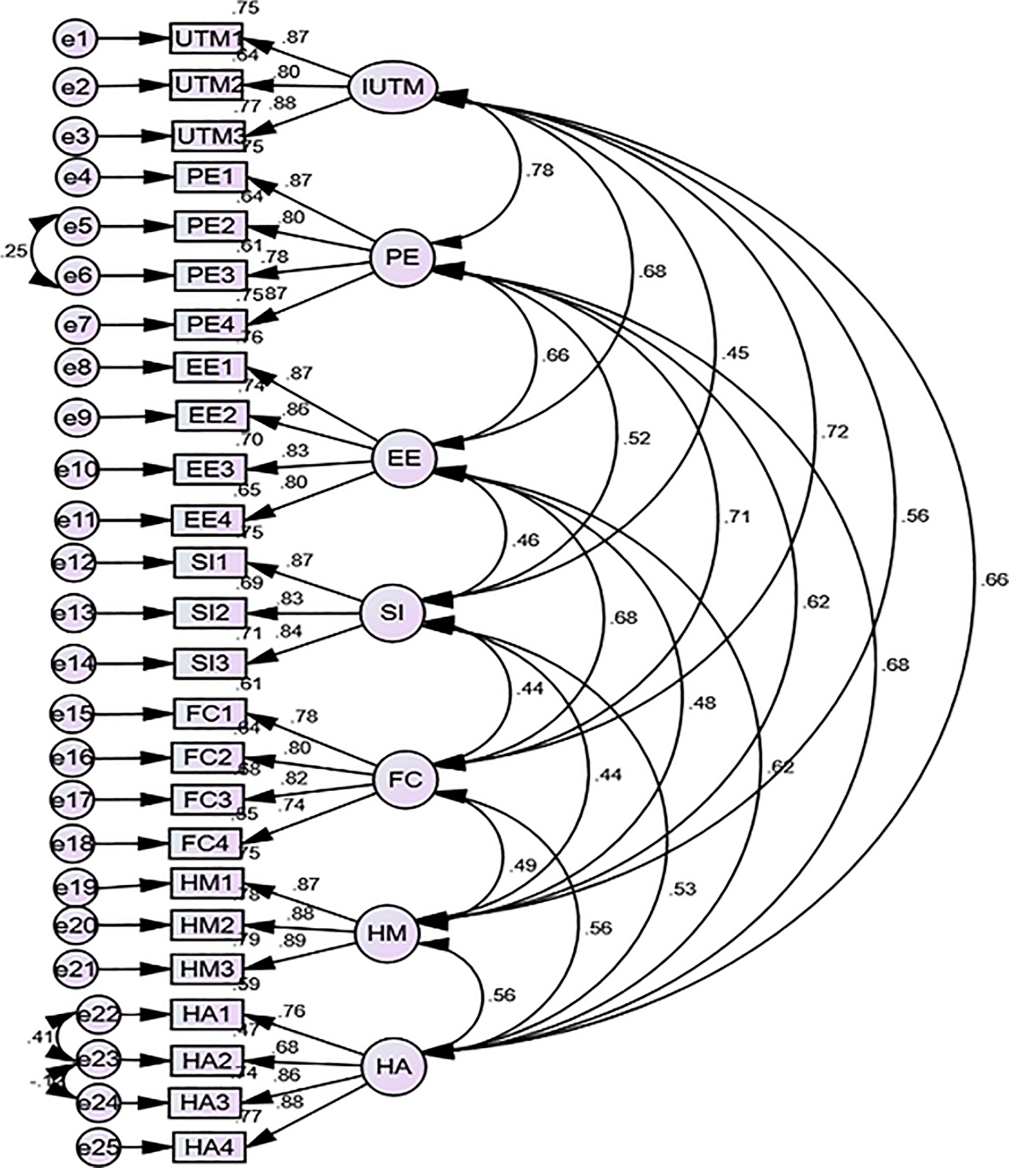
Confirmatory factor analysis among nurses and physicians at public hospitals in Bahir Dar, northwest Ethiopia, 2023.

Assessment of the measurement model involves checking the model goodness of fit, internal consistency (reliability), convergent validity, discriminant validity, and Kaiser–Meyer–Olkin (KMO) of indicators/items using CFA. To improve the model fit, covariate error terms with high modification indices were applied depending on their respective highest modification indices. I allowed a covariate between e5 with e6, e22 with e23, and e23 with e24 (Figure 3).

In this study, the correlation between each item was less than 0.8, the multivariate critical ratio did not range between −1.69 and +1.69 [critical ratio (CR) = 30.826], and the multivariate kurtosis value was not <5 (kurtosis = 83.725). The data were not normally distributed. In this case, the non-parametric test of bootstrapping methods was applied to non-normal data by resampling the data that assume a normal distribution, and it estimates the significance of the path coefficients, standard errors, and confidence intervals (54, 55). Thus, 5,000 bootstrap samples of 95% bias-corrected confidence interval in AMOS were applied. The relationship between each exogenous and endogenous variable was linear (*p* < 0.001).

### Reliability and validity of the construct

The AVE values for all constructs were found to be above 0.622, which was accepted to test convergent validity. The square root AVE values in bold (diagonal values) were higher than other values in its column, and row, and the HTMT ratio was less than 0.79. As a result, the discriminant validity of the model constructs was achieved (Table 2).

**Table 2:** Discriminant and convergent validity among nurses and physicians at public hospitals in Bahir Dar, northwest Ethiopia, 2023.

	AVE	IUTM	PE	EE	SI	FC	HM	HA
IUTM	0.720	**0** **.** **849**						
PE	0.687	0.784	**0**.**829**					
EE	0.711	0.680	0.661	**0**.**843**				
SI	0.716	0.454	0.516	0.464	**0**.**846**			
FC	0.622	0.722	0.714	0.677	0.442	**0**.**789**		
HM	0.772	0.556	0.615	0.475	0.437	0.493	**0**.**879**	
HA	0.638	0.665	0.678	0.621	0.533	0.561	0.652	**0.799**

AVE, average variance extracted.

The bold value provides that the, AVE.

Factor loadings for each item were investigated, and the value of factor loading for each item was found to be more than 0.68. The reliability test done by using composite reliability had values above 0.83 for all constructs (Table 3).

**Table 3:** Reliability test among nurses and physicians at public hospitals in Bahir Dar, northwest Ethiopia, 2023.

Construct	Indicators	Factor loading	Composite reliability
IUTM	IUTM1	0.87	0.885
	IUTM2	0.80	
	IUTM3	0.88	
Performance expectancy	PE1	0.87	0.898
	PE2	0.80	
	PE3	0.78	
	PE4	0.87	
Effort expectancy	EE1	0.87	0.908
	EE2	0.86	
	EE3	0.83	
	EE4	0.80	
Social influence	SI1	0.87	0.883
	SI2	0.83	
	SI3	0.84	
Facilitating condition	FC1	0.78	0.868
	FC2	0.80	
	FC3	0.82	
	FC4	0.74	
Hedonic motivation	HM1	0.87	0.910
	HM2	0.88	
	HM3	0.89	
Habit	HA1	0.76	0.875
	HA2	0.68	
	HA3	0.86	
	HA4	0.88	

### KMO test

Before performing factor analysis, it is recommended to perform the KMO test. The KMO test is a statistical measure to determine how suited data are for factor analysis. The KMO test measures sampling adequacy for each variable and overall variable. In this model, the values of the KMO test were 0.738, 0.833, 0.840, 0.746, 0.827, 0.756, and 797 for IUTM, PE, EE, SI, FC, HM, and HA, respectively, and the overall model value was 0.945. Because the KMO values were greater than 0.5, it is indicated that the research sample was sufficient to carry out factor analysis (56).

### Goodness of fit

The values of the model goodness of fit were assessed and met the required level (Table 4).

**Table 4:** Model fit indices among nurses and physicians at public hospitals in Bahir Dar, northwest Ethiopia, 2023.

Measure	Estimate	Threshold	Interpretation
GFI	0.931	>0.9	Supported
AGFI	0.911	>0.8	Supported
Chi-square/degree of freedom	2.527	<3	Supported
Comparative fit index (CFI)	0.972	>0.9	Supported
Standardized root mean squared residual (SRMR)	0.036	<0.08	Supported
RMSEA	0.046	<0.08	Supported

### Structural equation model assessment

Following the assessment of the measurement model, collinearity was evaluated along with model validity, reliability, fitness, and normalcy. Tolerance and the VIF were used to measure the degree of collinearity. When VIF and tolerance are less than 10 and more than 0.1, respectively, multicollinearity does not occur. In this analysis, multicollinearity was shown to be non-existent (Table 5).

**Table 5:** Multicollinearity test among nurses and physicians at public hospitals in Bahir Dar, northwest Ethiopia, 2023.

Exogenous construct	Collinearity statistics	
	Tolerance	Variance inflation factor
PE	0.265	3.774
EE	0.366	2.733
SI	0.607	1.646
FC	0.328	3.051
HM	0.520	1.923
HA	0.427	2.341

The SEM analysis shows that performance expectancy, effort expectancy, social influence, facilitating condition, hedonic motivation, and habit 69.4% (R-square = 0.694) with 95.0 CI of 62.5–74.9 explained the intention to use telemonitoring among nurses and physicians.

**Figure 4:**
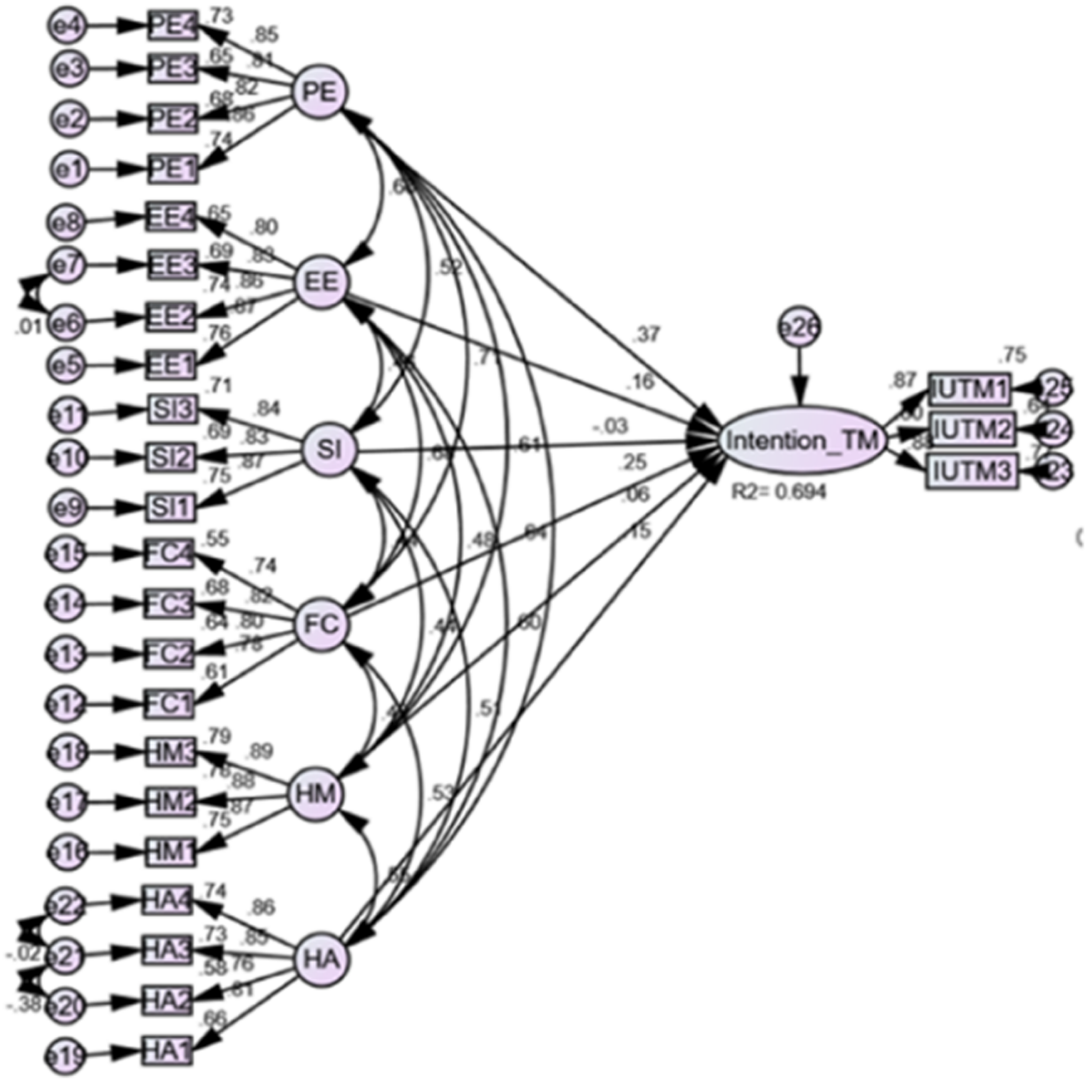
SEM for the predictors of intention to use telemonitoring among nurses and physicians at public hospitals in Bahir Dar, northwest Ethiopia, 2023.

According to the model's path coefficient, performance expectation and facilitating condition are the most powerful significant factors of intention to use telemonitoring (PE: *β* = 0.375, 95% CI: 0.258–0.494; FC: *β* = 0.255, 95% CI: 0.144–0.368). Effort expectancy has a direct positive effect on the intention to use telemonitoring (*β* = 0.158, 95% CI: 0.058–0.252). Habit also has a direct positive effect on the intention to use telemonitoring (*β* = 0.147, 95% CI: 0.059–0.233) (Table 6, Figure 4).

**Table 6:** SEM analysis of predictors of intention to use telemonitoring among nurses and physicians at public hospitals in Bahir Dar, northwest Ethiopia, 2023.

Hypothesis	Estimate	SE	*p*-value	95% CI		Result
				Lower	Upper	
PE → intention to use TM (H1)	0.375	0.051	0.001	0.258	0.494	Supported
EE → intention to use TM (H2)	0.158	0.040	0.003	0.058	0.252	Supported
SI → intention to use TM (H3)	−0.028	0.034	0.434	−0.095	0.039	Not supported
FC → intention to use TM (H4)	0.255	0.048	0.000	0.144	0.368	Supported
HM → intention to use TM (H5)	0.057	0.036	0.159	−0.024	0.137	Not supported
HA → intention to use TM (H6)	0.147	0.044	0.001	0.059	0.233	Supported

SE, standard error; CI, confidence interval.

### Testing potential moderators

In this finding, the moderation effects of gender and age of study participants with the relationship between performance expectancy, effort expectancy, social influence, facilitating condition, hedonic motivation, and habit with the intention to use telemonitoring to support chronic patients were investigated.

To test whether the moderator exists or not, the two-model comparison, which includes unconstrained and constrained (structural weight) models, was applied. The structural weight model assumption showed that there is a moderator or in the given variable to influence the exogenous and endogenous variables, whereas the unconstrained model suggests that the variable has a different effect on influencing the relationship between exogenous and endogenous variables. If the difference between the two models was found to be significant (a *p*-value < 0.05 or chi-square difference > 5), then the proposed moderator variable was confirmed as a moderator (49).

### Gender moderators’ test

According to this study, the overall effects of gender as a moderator were found to be significant with a chi-square ratio of 14.442 and *p*-values of 0.025. Testing the moderator effect of gender from each construct, it was found that only effort expectancy had a positive influence on the intention to use telemonitoring, which was positively moderated by gender and significantly stronger for male respondents (*β* = 0.311, *p*-value < 0.001) compared with that for female respondents (*β* = 0.025, *p*-value = 0.648), with a chi-square ratio of 10.461 and a *p*-value of 0.001 model comparations. On the other hand, performance expectancy, social influence, facilitating conditions, habit, and hedonic motivation on the intention to use telemonitoring were not significantly different between individuals by gender (Table 7).

**Table 7:** Moderating effects of gender on intention to use telemonitoring among nurses and physicians at public hospitals in Bahir Dar, northwest Ethiopia, 2023.

Hypothesis	Gender as moderator	Path coefficient	*p*-value	Model test (unconstrained and constrained model)		Result
				ΔX^2^	*p*-value	
PE → intention to use TM	Female	0.425	0.000	0.756	0.384	Not support
	Male	0.338	***			
EE → intention to use TM	Female	0.025	0.648	10.461	0.001	Support
	Male	0.311	***			
SI → intention to use TM	Female	−0.062	0.184	0.670	0.413	Not support
	Male	−0.008	0.866			
FC → intention to use TM	Female	0.362	***	0.715	0.398	Not support
	Male	0.261	0.003			
HM → intention to use TM	Female	0.070	0.130	0.186	0.666	Not support
	Male	0.042	0.354			
HA → intention to use TM	Female	0.136	0.012	0.015	0.902	Not support
	Male	0.146	0.014			

*** Significance at *p* < 0.001.

### Age moderators’ test

According to this study, the overall effects of age as a moderator were found to be significant with a chi-square ratio of 26.843 and *p*-values of <0.001. Testing the moderator effect of age from each construct effort expectancy and facilitating condition had a positive influence on the intention to use telemonitoring. Effort expectancy was positively moderated by age and significantly stronger for young respondents (*β* = 0.372, *p*-value < 0.001) compared with that for adult respondents (*β* = 0.087, *p*-value < 0.089), with a chi-square ratio of 7.486 and a *p*-value of 0.006 model comparations. The facilitating condition was also positively moderated by age and significantly stronger for adult respondents (*β* = 0.421, *p*-value < 0.001) compared with that for young respondents (*β* = −0.046, *p*-value < 0.671), with a chi-square ratio of 12.420 and a *p*-value of <0.001 model comparations. On the other hand, performance expectancy, social influence, habit, and hedonic motivation on the intention to use telemonitoring were not significantly different between individuals by age (Table 8).

**Table 8:** Moderating effects of age on intention to use telemonitoring among nurses and physicians at public hospitals in Bahir Dar, northwest Ethiopia, 2023.

Hypothesis	Gender as moderator	Path coefficient	*p*-value	Model test (unconstrained and constrained model)		Result
				ΔX^2^	*p*-value	
PE → intention to use TM	Young	0.431	***	0.478	0.489	Not support
	Adult	0.356	***			
EE → intention to use TM	Young	0.372	***	7.486	0.006	Support
	Adult	0.087	0.089			
SI → intention to use TM	Young	−0.114	0.069	3.548	0.060	Not support
	Adult	0.025	0.521			
FC → intention to use TM	Young	−0.046	0.671	12.420	0.000	Support
	Adult	0.421	***			
HM → intention to use TM	Young	0.030	0.593	0.089	0.765	Not support
	Adult	0.051	0.189			
HA → intention to use TM	Female	0.287	***	3.358	0.067	Not support
	Male	0.115	0.015			

*** Significance at *P* < 0.001.

## Discussion

This study investigates the intention to use telemonitoring and its associated factors among nurses and physicians at public hospitals in Bahir Dar, northwest Ethiopia. In resource-limited settings, they face scarce technological infrastructure and have low socioeconomic status to adopt the new technology. Telemonitoring is at its infancy; however, this study revealed that the intention of nurses and physicians to use telemonitoring was promising.

This finding can be rated higher than that of a study done at public hospitals in Iluababor and Buno Bedele zones, Oromia Region, southwest Ethiopia, in 2021 on the willingness to use telemedicine, which showed a rate of 46.5% (19). The sample size of this study was small, and roughly one-third of the participants had diplomas, which may have implications for using smart devices and the Internet, coupled with the fact that this study included healthcare workers from rural hospitals who may have limited access to electricity and the Internet (19). These factors could be reasons for the discrepancy in the rates. Also, Bahir Dar was a more urbanized urban area compared with the area of this study. Another possible reason could be that about half of the study participants had an average monthly income below five thousand Ethiopian birrs, which could have implications for affording Internet and new technology (19).

The current proposed model explains a 69.4% variance (*R*^2^ = 0.694) in the intention of nurses and physicians to use telemonitoring to support chronic patients at public hospitals in Bahir Dar, northwest Ethiopia. This means that all exogenous variables strongly predicted the intention to use telemonitoring. The intention to use telemonitoring was significantly associated with performance expectancy, effort expectancy, facilitating condition, and habit, indicating that four out of six path relationships in the proposed model were directly associated with the intention to employ telemonitoring. Accordingly, H1, H2, H4, and H6 were supported. Based on the results of this study, the following observations are given to describe the enhanced intention to use telemonitoring by nurses and physicians at public hospitals in Bahir Dar City, northwest Ethiopia.

Performance expectancy had a direct effect on nurses’ and physicians’ intention to use telemonitoring. This indicates that nurses and physicians are motivated to use telemonitoring to support chronic patients when this technology improves their work performances and quality of services, useful for their work, and applies their tasks more quickly. Therefore, a net positive effect from performance expectancy will result in a positive effect on the intention to use telemonitoring. This result is consistent with that of previous studies done in Ethiopia (31), Bilbao Primary Care (20) and Don Ostia University Hospital (24), Spain, China (57), India (27), and Indonesia (35). The possible reason for this could be the usefulness of telemonitoring to improve the quality and performance of healthcare deliveries (58, 59). This finding contrasts with the study done in Ethiopia (32). The reason for this discrepancy could be sufficiently biased to be interested in the subject due to data collected in an online survey and the use of a smaller sample size in the previous study (32).

The finding has illustrated that effort expectancy had a direct effect on nurses and physicians’ intention to use telemonitoring. This showed that their intention to use telemonitoring will be enhanced when it is easy to learn, easy to perform tasks, interaction with them is clear and understandable, and easy to become skillful. This result is consistent with that of other studies conducted in Ethiopia (32), India (27), and Indonesia (33). The possible reason for this may be that nowadays, nurses and physicians are exposed to the practice of day-to-day use of information technologies. Currently, sophisticated technologies are emerging, and remote healthcare is delivered with little effort (2). This finding contrasts with that of another study done in Don Ostia University Hospital (24). The possible reason for this could be the time spent doing the research, which is outdated with a smaller sample size (24).

Similarly, facilitating conditions had a direct positive effect on the intention to telemonitoring. The result revealed that the availability of resources, support, compatibility with other technologies, and knowledge is necessary to motivate nurses and physicians to use telemonitoring. This is consistent with that of studies conducted in Ethiopia (31), Spain, (20), (24), Indonesia (33), China (58), and India (27). The possible reason for this could be the significance of providing health professionals with organizational and technological infrastructure and its impact on the intention of health professionals to use telemonitoring.

Habit also has a direct positive effect on the intention to use telemonitoring; with every increase in habit, the intention to use telemonitoring also increases. This shows that when the use of telemonitoring becomes a habit, users would be naturally addicted to it, thus paving the way for enhanced intention to use it. This result is consistent with that of other similar studies in the United States (39). The possible explanation for this could be that due to an individual's belief, frequent use of technology leads to an automatic behavior (60) to use it.

The findings indicate that effort expectancy had a positive influence on the intention to use telemonitoring, which was positively moderated by gender and significantly stronger for male respondents compared with that for female respondents. This is consistent with the research conducted in India (27) and the original model (23). The possible reason could be women are expected to be less interested in and less capable of using technology compared with men as they may be busy with their household work and men may earn more money to afford its use (61). Another possible explanation could be males hold a more favorable attitude toward technology use than women (62). As a result, men believe that using telemonitoring is easy.

The effect of effort expectancy on the intention to use telemonitoring was positively moderated by age and significantly stronger for young respondents compared with that for adult respondents. This is consistent with the original model (23). The possible reason could be younger members of society engage more with technologies, and with increased age, individuals might show a decline in psychomotor skills, processing capacity, and increased distraction when using technologies (63). This finding contrasts with a study done in India (27). The possible reason for this discrepancy could be adult participants were more experienced with the system, and therefore, they used TM with little effort (27).

The relationship between facilitating conditions and intention to use telemonitoring was positively moderated by age and significantly stronger for adult respondents compared with that for young respondents. This finding is consistent with that of studies conducted in India (27) and the original model (23). The possible reason could be that compared with younger adults, adult respondents tend to place greater importance on the availability of adequate support (23, 63).

### Implications

The study highlights significant implications for the intention to use telemonitoring in managing chronic diseases among nurses and physicians, particularly through the lens of the UTAUT2 model. It emphasizes performance expectancy, showing that telemonitoring enhances patient engagement and adherence to treatment by facilitating regular communication and self-monitoring, which can lead to improved health outcomes through timely interventions and personalized care.

With regard to effort expectancy, telemonitoring streamlines care by reducing the need for in-person visits, thus improving efficiency and necessitating training for healthcare providers to effectively use the technology. This training promotes collaboration and knowledge sharing within healthcare teams.

This study underscores the significant influence of habit formation on the intention to utilize telemonitoring for the management of chronic diseases by facilitating regular engagement with telemonitoring tools; healthcare providers can assist patients in establishing consistent usage patterns. Evidence-based strategies such as automated reminders, user-friendly interfaces, and structured peer support can effectively promote these habits. As telemonitoring becomes integrated into the patients’ daily health management practices, their intention to adopt such technologies is expected to increase, ultimately contributing to enhanced health outcomes and improved adherence to treatment regimens. In addition, the findings support facilitating conditions, advocating for a patient-centered care model that tailors treatment plans to individual clinical and lifestyle needs. This research may also guide policy decisions on telehealth guidelines and funding, ensuring adequate support for effective chronic disease management.

Overall, the study underscores the transformative potential of telemonitoring in improving patient management and health outcomes, reinforcing the importance of UTAUT2 constructs in fostering the adoption of telemonitoring technologies.

## Conclusion

In conclusion, more than half of the nurses and physicians working at public hospitals in Bahir Dar of northwest Ethiopia intended to use telemonitoring. Performance expectancy, effort expectancy, facilitating conditions, and habit were statistically significant factors of intention to use telemonitoring. Among the four exogenous variables, performance expectancy had a more significant prediction power of study participants’ intention to use telemonitoring. Age and gender positively moderated the link between effort expectancy and intention to employ telemonitoring; young people and men were strongly associated, respectively. Age positively attenuated the association between the intention to use telemonitoring and the facilitating conditions, and the adult age group was strongly associated with the relationship.

### Limitations of the study

This study does not show causality, only establishing correlations between variables, and therefore has the limitation of a cross-sectional study. The research sample that was used was taken only from public hospitals in Bahir Dar, northwest Ethiopia, which affects the generalizability of the findings.

## Data Availability

The raw data supporting the conclusions of this article will be made available by the authors without undue reservation.

## References

[1] Thrall JH Boland G. Telemedicine in Practice. in Seminars in Nuclear Medicine. Boston: Elsevier (1998). 10.1016/s0001-2998(98)80004-4 9579416

[2] Haleem A Javaid M Singh RP Suman R. Telemedicine for healthcare: capabilities, features, barriers, and applications. Sens Int. (2021) 2:100117. 10.1016/j.sintl.2021.100117 34806053 PMC8590973

[3] Masuet AN. Exploring Telemedicine: a comprehensive overview of telemedicine, telemonitoring, and technologies for remote health and hypertension monitoring. (2023).

[4] Losiouk E Lanzola G Del Favero S Boscari F Messori M Rabbone I Parental evaluation of a telemonitoring service for children with type 1 diabetes. J Telemed Telecare. (2018) 24(3):230–7. 10.1177/1357633X17695172 28345384

[5] Al-Ofi EA Mosli HH Ghamri KA Ghazali SM. Management of postprandial hyperglycaemia and weight gain in women with gestational diabetes mellitus using a novel telemonitoring system. J Int Med Res. (2019) 47(2):754–64. 10.1177/0300060518809872 30442052 PMC6381491

[6] Margolis KL Asche SE Dehmer SP Bergdall AR Green BB Sperl-Hillen JM Long-term outcomes of the effects of home blood pressure telemonitoring and pharmacist management on blood pressure among adults with uncontrolled hypertension: follow-up of a cluster randomized clinical trial. JAMA Network Open. (2018) 1(5):e181617–e181617. 10.1001/jamanetworkopen.2018.1617 30646139 PMC6324502

[7] Wild SH Hanley J Lewis SC McKnight JA McCloughan LB Padfield PL Supported telemonitoring and glycemic control in people with type 2 diabetes: the telescot diabetes pragmatic multicenter randomized controlled trial. PLoS Med. (2016) 13(7):e1002098. 10.1371/journal.pmed.1002098 27458809 PMC4961438

[8] Nangalia V Prytherch DR Smith GB. Health technology assessment review: remote monitoring of vital signs–current status and future challenges. Crit Care. (2010) 14(5):233. 10.1186/cc9208 20875149 PMC3219238

[9] Mucchi L Jayousi S Gant A Paoletti E Zoppi P. Tele-monitoring system for chronic diseases management: requirements and architecture. Int J Environ Res Public Health. (2021) 18(14):7459. 10.3390/ijerph18147459 34299910 PMC8305785

[10] Yach D Hawkes C Gould CL Hofman KJ. The global burden of chronic diseases: overcoming impediments to prevention and control. JAMA. (2004) 291(21):2616–22. 10.1001/jama.291.21.2616 15173153

[11] Mariye T Tasew H Teklay G Gerensea H Daba W. Magnitude of diabetes self-care practice and associated factors among type two adult diabetic patients following at public hospitals in central zone, Tigray Region, Ethiopia, 2017. BMC Res Notes. (2018) 11(1):380. 10.1186/s13104-018-3489-0 29895315 PMC5998566

[12] Tewahido D Berhane Y. Self-Care practices among diabetes patients in Addis Ababa: a qualitative study. PLoS One. (2017) 12(1):e0169062. 10.1371/journal.pone.0169062 28045992 PMC5207399

[13] Li J Varnfield M Jayasena R Celler B. Home telemonitoring for chronic disease management: perceptions of users and factors influencing adoption. Health Informatics J. (2021) 27:146045822199789. 10.1177/1460458221997893 33685279

[14] Viderman D Seri E Aubakirova M Abdildin Y Badenes R Bilotta F. Remote monitoring of chronic critically ill patients after hospital discharge: a systematic review. J Clin Med. (2022) 11(4):1010. 10.3390/jcm11041010 35207287 PMC8879658

[15] Huygens MW Voogdt-Pruis HR Wouters M Meurs MM Van Lettow B Kleijweg C The uptake and use of telemonitoring in chronic care between 2014 and 2019: nationwide survey among patients and health care professionals in The Netherlands. J Med Internet Res. (2021) 23(5):e24908. 10.2196/24908 33938808 PMC8129877

[16] MOHE. National Digital Health Strategy. (2020-2029).

[17] Ogundaini O de la Harpe R McLean N. Unintended consequences of technology-enabled work activities experienced by healthcare professionals in tertiary hospitals of sub-Saharan Africa. Af J Sci Technol Innov Develop. (2022) 14(4):876–85. 10.1080/20421338.2021.1899556

[18] Manyazewal T Woldeamanuel Y Blumberg HM Fekadu A Marconi VC. The potential use of digital health technologies in the African context: a systematic review of evidence from Ethiopia. NPJ Digital Medicine. (2021) 4(1):125. 10.1038/s41746-021-00487-4 34404895 PMC8371011

[19] Ahmed MH Awol SM Kanfe SG Hailegebreal S Debele GR Dube GN Willingness to use telemedicine during COVID-19 among health professionals in a low income country. Inform Med Unlocked. (2021) 27:100783. 10.1016/j.imu.2021.100783 34778509 PMC8571100

[20] Asua J Orruño E Reviriego E Gagnon MP. Healthcare professional acceptance of telemonitoring for chronic care patients in primary care. BMC Med Inform Decis Mak. (2012) 12:139. 10.1186/1472-6947-12-139 23194420 PMC3520721

[21] Walker RC Tong A Howard K Palmer SC. Patient expectations and experiences of remote monitoring for chronic diseases: systematic review and thematic synthesis of qualitative studies. Int J Med Inf. (2019) 124:78–85. 10.1016/j.ijmedinf.2019.01.013 30784430

[22] Venkatesh V Morris MG Davis GB Davis FD. User acceptance of information technology: toward a unified view. MIS Q. (2003) 27:425–78. 10.2307/30036540

[23] Venkatesh V Thong JY Xu X. Consumer acceptance and use of information technology: extending the unified theory of acceptance and use of technology. MIS Q. (2012) 36:157–78. 10.2307/41410412

[24] Gagnon MP Orruño E Asua J Abdeljelil AB Emparanza J. Using a modified technology acceptance model to evaluate healthcare professionals’ adoption of a new telemonitoring system. Telemed J E Health. (2012) 18(1):54–9. 10.1089/tmj.2011.0066 22082108 PMC3270047

[25] Pecina JL Vickers KS Finnie DM Hathaway JC Takahashi PY Hanson GJ. Health care providers style may impact acceptance of telemonitoring. Home Health Care Manag Pract. (2012) 24(6):276–82. 10.1177/1084822312440706

[26] Özdemir-Güngör D Camgöz-Akdağ H. Examining the effects of technology anxiety and resistance to change on the acceptance of breast tumor registry system: evidence from Turkey. Technol Soc. (2018) 54:66–73. 10.1016/j.techsoc.2018.03.006

[27] Seethamraju R Diatha KS Garg S. Intention to use a mobile-based information technology solution for tuberculosis treatment monitoring–applying a UTAUT model. Information Systems Frontiers. (2018) 20(1):163–81. 10.1007/s10796-017-9801-z

[28] Rouidi M Hamdoune A Choujtani K Chati A. TAM-UTAUT and the acceptance of remote healthcare technologies by healthcare professionals: a systematic review. Inform Med Unlocked. (2022) 101008. 10.1016/j.imu.2022.101008

[29] Ammenwerth E. Technology acceptance models in health informatics: TAM and UTAUT. Stud Health Technol Inform. (2019) 263:64–71. 10.3233/SHTI190111 31411153

[30] Hoque MR Bao Y. Cultural influence on adoption and use of e-health: evidence in Bangladesh. Telemed E-Health. (2015) 21(10):845–51. 10.1089/tmj.2014.0128 26348844

[31] Kalayou MH Endehabtu BF Tilahun B. The applicability of the modified technology acceptance model (TAM) on the sustainable adoption of eHealth systems in resource-limited settings. J Multidiscip Healthc. (2020) 13:1827–37. 10.2147/JMDH.S284973 33299320 PMC7721313

[32] Shiferaw KB Mengiste SA Gullslett MK Zeleke AA Tilahun B Tebeje T Healthcare providers’ acceptance of telemedicine and preference of modalities during COVID-19 pandemics in a low-resource setting: an extended UTAUT model. PLoS One. (2021) 16(4):e0250220. 10.1371/journal.pone.0250220 33886625 PMC8061916

[33] Napitupulu D Yacub R Putra A. Factor influencing of telehealth acceptance during COVID-19 outbreak: extending UTAUT model. Int J Intel Engineer Syst. (2021) 14(3):267–81. 10.22266/ijies2021.0630.23

[34] Lee Y Kozar KA Larsen KR. The technology acceptance model: past, present, and future. Commun Assoc Inf Syst. (2003) 12(1):50. 10.17705/1CAIS.01250

[35] Kuo KM Talley PC Lee CM Yen YC. The influence of telemedicine experience on physicians’ perceptions regarding adoption. Telemed E-Health. (2015) 21(5):388–94. 10.1089/tmj.2014.0091 25764024

[36] Ly BA Kristjansson E Labonte R Bourgeault IL. Determinants of the intention of Senegal’s physicians to use telemedicine in their professional activities. Telemed E-Health. (2018) 24(11):897–8. 10.1089/tmj.2017.0276 29470109

[37] Shi J Yan X Wang M Lei P Yu G. Factors influencing the acceptance of pediatric telemedicine services in China: a cross-sectional study. Front Pediatr. (2021) 9:745687. 10.3389/fped.2021.745687 34733810 PMC8558490

[38] Rahi S. Assessing individual behavior towards adoption of telemedicine application during COVID-19 pandemic: evidence from emerging market. Library Hi Tech. (2022) 40(2):394–420. 10.1108/LHT-01-2021-0030

[39] Yuan S Ma W Kanthawala S Peng W. Keep using my health apps: discover users’ perception of health and fitness apps with the UTAUT2 model. Telemed E-Health. (2015) 21(9):735–41. 10.1089/tmj.2014.0148 25919238

[40] Beh PK Ganesan Y Iranmanesh M Foroughi B. Using smartwatches for fitness and health monitoring: the UTAUT2 combined with threat appraisal as moderators. Behav Inf Technol. (2021) 40(3):282–99. 10.1080/0144929X.2019.1685597

[41] Lorella C Rippa P Caputo S. User acceptance of healthcare robots through extended UTAUT2: a mixed method approach. *Research Square* [Preprint]. (2022). 10.21203/rs.3.rs-2326113/v1

[42] Chang A. UTAUT And UTAUT 2: a review and agenda for future research. The Winners. (2012) 13(2):10–114. 10.21512/tw.v13i2.656

[43] Zhao Y Ni Q Zhou R. What factors influence the mobile health service adoption? A meta-analysis and the moderating role of age. Int J Inf Manage. (2018) 43:342–50. 10.1016/j.ijinfomgt.2017.08.006

[44] Xiong X Mei Q. Study on the factors influencing user’s acceptance intention for smart medical and health care equipment based on UTAUT2. DEStech transactions on economics. Bus Manag. (2016). 10.12783/dtssehs/apme2016/8110

[45] ABOFED, population of Bahir Dar city (2018).

[46] Kline RB. Principles and Practice of Structural Equation Modeling. New York, NY: Guilford Publications (2015).

[47] Wang YA Rhemtulla M. Power analysis for parameter estimation in structural equation modeling: a discussion and tutorial. Adv Methods Pract Psycho Sci. (2021) 4(1):2515245920918253. 10.1177/2515245920918253

[48] Weston R Gore PA Jr. A brief guide to structural equation modeling. Couns Psychol. (2006) 34(5):719–51. 10.1177/0011000006286345

[49] Walle AD Jemere AT Tilahun B Endehabtu BF Wubante SM Melaku MS Intention to use wearable health devices and its predictors among diabetes mellitus patients in Amhara region referral hospitals, Ethiopia: using modified UTAUT-2 model. Inform Med Unlocked. (2022) 36:101157. 10.1016/j.imu.2022.101157

[50] Ahmed MH Bogale AD Tilahun B Kalayou MH Klein J Mengiste SA et al. Intention to use electronic medical record and its predictors among health care providers at referral hospitals, north-west Ethiopia, 2019: using unified theory of acceptance and use technology 2 (UTAUT2) model. BMC Med Inform Decis Mak. (2020) 20(1):1–11. 10.1186/s12911-020-01222-x 32883267 PMC7469309

[51] Kharuddin AF Azid N Mustafa Z Ibrahim KF Kharuddin D. Application of structural equation modeling (SEM) in estimating the contributing factors to satisfaction of TASKA services in east coast Malaysia. Asian J Assess Teach Learn. (2020) 10(1):69–77. 10.37134/ajatel.vol10.1.8.2020

[52] Mustafa M Nordin M Razzaq A. Structural equation modelling using AMOS: confirmatory factor analysis for taskload of special education integration program teachers. Univers J Educ Res. (2020) 8(1):127–33. 10.13189/ujer.2020.080115

[53] Sidelil H Demissie A Demissie GD Fikade B Hailegebreal S Tilahun B. Attitude towards tele rehabilitation-based therapy services and its associated factors among health professional working in specialized teaching hospitals in Amhara region, Northwest Ethiopia. Inform Med Unlocked. (2023) 36:101145. 10.1016/j.imu.2022.101145

[54] Hu C Wang Y. Bootstrapping in AMOS. Powerpoint. Consulté le, (2010): p. 23-02.

[55] Purwaningsih R Sekarini DA Susanty A Pramono SN. The influence of bootstrapping in testing a model of motivation and visit intention of generation Z to the attractive building architecture destinations. IOP Conference Series: Earth and Environmental Science (2021). IOP Publishing.

[56] Taufan A Yuwono RT. Analysis of factors that affect intention to use e-wallet through the technology acceptance model approach (case study: GO-PAY). Int J Sci Res. (2019) 8(7):413–9. 10.21275/ART2020219

[57] Pan M Gao W. Determinants of the behavioral intention to use a mobile nursing application by nurses in China. BMC Health Serv Res. (2021) 21(1):228. 10.1186/s12913-021-06244-3 33712012 PMC7953719

[58] Taylor ML Thomas EE Snoswell CL Smith AC Caffery LJ. Does remote patient monitoring reduce acute care use? A systematic review. BMJ Open. (2021) 11(3):e040232. 10.1136/bmjopen-2020-040232 33653740 PMC7929874

[59] Tomasic I Tomasic N Trobec R Krpan M Kelava T. Continuous remote monitoring of COPD patients—justification and explanation of the requirements and a survey of the available technologies. Med Biol Eng Comput. (2018) 56:547–69. 10.1007/s11517-018-1798-z 29504070 PMC5857273

[60] Kim Y Zhang P. Continued use of technology: combining controlled and automatic processes. *ICIS 2010 Proceedings*. (2010):214.

[61] Sobieraj S Krämer NC. Similarities and differences between genders in the usage of computer with different levels of technological complexity. Comput Human Behav. (2020) 104:106145. 10.1016/j.chb.2019.09.021

[62] Cai Z Fan X Du J. Gender and attitudes toward technology use: a meta-analysis. Comput Educ. (2016) 105:1–13. 10.1016/j.compedu.2016.11.003

[63] Slade EL Williams M Dwivedi Y. An extension of the UTAUT 2 in a healthcare context. *UK Academy for Information Systems Conference Proceedings*. (2013). p. 55.

